# Risk factors in the development of stem cell therapy

**DOI:** 10.1186/1479-5876-9-29

**Published:** 2011-03-22

**Authors:** Carla A Herberts, Marcel SG Kwa, Harm PH Hermsen

**Affiliations:** 1 Centre for Biological Medicines and Medical Technology, National Institute for Public Health and the Environment, A. v. Leeuwenhoeklaan 9, P.O.Box 1, 3720 BA, Bilthoven, The Netherlands; 2 Department of Pharmacovigilance, Netherlands Medicines Evaluation Board, Kalvermarkt 53, 2511 CB, Den Haag, The Netherlands

**Keywords:** stem cell therapy, advanced therapy, risk, medicinal product, stem cells, tumour, immunology, clinical, ESC, iPSC, SSC, cell based medicinal product

## Abstract

Stem cell therapy holds the promise to treat degenerative diseases, cancer and repair of damaged tissues for which there are currently no or limited therapeutic options. The potential of stem cell therapies has long been recognised and the creation of induced pluripotent stem cells (iPSC) has boosted the stem cell field leading to increasing development and scientific knowledge. Despite the clinical potential of stem cell based medicinal products there are also potential and unanticipated risks. These risks deserve a thorough discussion within the perspective of current scientific knowledge and experience. Evaluation of potential risks should be a prerequisite step before clinical use of stem cell based medicinal products.

The risk profile of stem cell based medicinal products depends on many risk factors, which include the type of stem cells, their differentiation status and proliferation capacity, the route of administration, the intended location, *in vitro *culture and/or other manipulation steps, irreversibility of treatment, need/possibility for concurrent tissue regeneration in case of irreversible tissue loss, and long-term survival of engrafted cells. Together these factors determine the risk profile associated with a stem cell based medicinal product. The identified risks (*i.e*. risks identified in clinical experience) or potential/theoretical risks (*i.e*. risks observed in animal studies) include tumour formation, unwanted immune responses and the transmission of adventitious agents.

Currently, there is no clinical experience with pluripotent stem cells (i.e. embryonal stem cells and iPSC). Based on their characteristics of unlimited self-renewal and high proliferation rate the risks associated with a product containing these cells (e.g. risk on tumour formation) are considered high, if not perceived to be unacceptable. In contrast, the vast majority of small-sized clinical trials conducted with mesenchymal stem/stromal cells (MSC) in regenerative medicine applications has not reported major health concerns, suggesting that MSC therapies could be relatively safe. However, in some clinical trials serious adverse events have been reported, which emphasizes the need for additional knowledge, particularly with regard to biological mechanisms and long term safety.

## Introduction

Stem cells are undifferentiated cells that have the capacity to proliferate in undifferentiated cells both *in vitro *and *in vivo *(self-renewal) and to differentiate into mature specialized cells.

The field of stem cell therapy is rapidly developing, and many clinical trials have been initiated exploring the use of stem/progenitor cells in the treatment of degenerative diseases and cancer and for the repair of damaged or lost tissues. Despite the great promise, there are still many questions regarding the safe application of stem cell therapy. In this paper we will focus on risks associated with stem cell therapy, based on both theoretical concerns and examples of adverse observations.

Based on their characteristics different stem cells types have been described (table 1). The distinctive feature of different stem cell types is based on the capability of the cells to differentiate along multiple lineages and produce derivatives of cell types of the three germ layers or to produce multiple cell types. Below different stem cell types are briefly described.

**Table 1:** Characteristics of different types of stem cells

ESC	iPSC	SSC
Derived from inner cell mass of blastocyst	Derived from somatic cells	Isolated from postnatal adult tissue

Allogenic material	Autologous or allogenic material	Autologous or allogenic material

Pluripotent	Pluripotent	Multipotent

Can differentiate in cell types of all three germ lineages	Can differentiate in cell types of all three germ lineages	Can differentiate in limited cell types depending on the tissue of origin

Ability to form chimeras	Ability to form chimeras (maybe more difficult than for ESCs)	Cannot form chimeras

Self-renewal	Self-renewal	Limited self-renewal

Require many steps to drive differentiation into the desired cell type	Require many steps to manufacture (e.g. genetic modification) and to drive differentiation into the desired cell type	Difficult to maintain in cell culture for long periods

High degree of proliferation once isolated	High degree of proliferation	Ease of access, yield and purification varies, depending on the source tissue

Indefinite growth	Indefinite growth	Limited lifespan (population doublings)

Production of endless number of cells	Production of endless number of cells	Production of limited number of cells
Chromosome length is maintained across serial passage	Chromosomes tend to shorten with ageing	Chromosomes tend to shorten with ageing

Significant teratoma risk	Significant teratoma risk	No teratoma risk

Serious ethical issues	No ethical issues	No ethical issues

Immuno-priviliged. Low level of MHC I and II (also in ESC-derived cells)	Not immuno-priviliged when derived from adult cells. Normal level of MHC I and II molecules.	MSC have low immunogenicity and are immunomodulatory. Not known for other somatic SC.

Cell lines will be allogenic	Less chance immune rejection in case of HLA-matching	In case of autologous use, less chance of immune rejection, but immunogenicity in allogenic and non-homologous applications remains unpredictable

Donor history may be unknown for 'old' cell lines (i.e. initially not intended for clinical application)	Targeted disease may still be present in stem cell in case of autologous use	Targeted disease may still be present in stem cell in case of autologous use

### Embryonal stem cells

In the early sixties researchers isolated a single cell type from a teratocarcinoma, a tumour derived from a germ cell. These embryonal carcinoma cells are the stem cells of teratocarcinomas which can be considered the malignant counterparts of embryonic stem cells that originate from the inner cell mass of a blastocyst stage embryo. The embryonal carcinoma cells replicate and grow in cell culture conditions.

In 1981, embryonic stem cells (ES cells) were first derived from mouse embryos [1,2]. Evans and Kaufman [1] revealed a new technique for culturing the mouse embryonic stem cells from embryos in the uterus to increase cell numbers, allowing for the derivation of ES cells from these embryos. Martin [2] showed that embryos could be cultured in vitro and that ES cells could be derived from these embryos. In 1998, Thomson et al [3] developed a technique to isolate and grow human embryonic stem cells in cell culture.

Embryonal stem cells (ESC) are pluripotent cells that have the ability to differentiate into derivatives of all three germ layers (endoderm, mesoderm, and ectoderm). The most common assay for demonstrating pluripotency is teratoma formation. However, pluripotent stem cell lines must be able to fulfil several other specific features [4,5]. Stem cell lines have the ability to grow indefinitely and express ESC markers and show ESC-like morphology. In addition, the cell line forms embryonic bodies (*in vitro*) and/or teratomas (*in vivo*) containing all 3 germlayers. In mice pluripotent stem cells have the ability to form chimeras upon injection into early blastocysts [5].

ESC are derived from totipotent cells of the inner cell mass of the blastocyst, an early stage mammalian embryo. These cells are capable of unlimited, undifferentiated proliferation in vitro [3]. In mouse embryo chimeras ESCs can differentiate into a range of adult tissues [6]. Also human ESCs have a large differentiation potential and can form cells from all embryonic germ layers [7]. In 1998 Thomson et al indicated that ESC cell lines were expected to become useful in drug discovery [3].

### Induced pluripotent stem cells

Induced pluripotent stem cells (iPSCs) are a type of pluripotent stem cells artificially derived from an adult differentiated somatic cell that is non-pluripotent. The transformation of an adult somatic cell into a pluripotent stem cell (iPSC) was firstly achieved by inducing a "forced" expression of specific genes [8-14]. At this moment it has been demonstrated that the forced expression of a characterized set of transcription factors (Oct4, Sox2, c-Myc, Klf4, Nanog, and Lin28) can reprogram human and mouse somatic cells into iPSCs [11,15]. Currently numerous alternative strategies for making iPSC have been reported, these will be discussed in this paper in a section on genetic modification.

For iPSC generation mostly fibroblasts are used, but iPSC have also been derived from liver, pancreas β cells and mature B cells [16]. Despite the difference in their origin, ESC and iPSC are very similar. They have highly similar growth characteristics, gene expression profiles, epigenetic modifications and developmental potential [16-18]. However, some differences in gene expression have been reported suggesting that reprogramming in iPSC is incomplete [17]. In addition, the generation of chimeras from iPSC appears more difficult than for ESCs and has been associated with a higher rate of tumour formation [19].

### Somatic stem cells

Multipotent somatic or adult stem cells (SSC) are found in differentiated tissues. The natural function of these cells is the maintenance and regeneration of aged or damaged tissue by replacing lost cells [20]. In general these undifferentiated cells are found throughout the body in juvenile as well as adult animals and humans. SSC can be subdivided into different groups, depending on their morphology, cell surface markers, differentiation potential, and/or tissue of origin. Examples are the mesenchymal stem/stromal cells (MSC), haematopoietic stem cells (HSC) and endothelial progenitor cells (EPS). Scientific interest in somatic or adult stem cells has centred on their ability to divide or self-renew indefinitely, and (with certain limitations) differentiate to yield all the specialized cell types of the tissue from which it originated. For example, neural stem cells are self-renewing multipotent cells that generate mainly phenotypes of the nervous system (e.g. neurons, astrocytes and oligodendrocytes) [21]. These cells play an important role in neurogenesis [22].

In principle SSC can be isolated from many tissues; however cord blood and bone marrow are sources which are often used as source of SSC for stem cell therapy. More recently, adipose tissue has also been used. Neural stem cells have been isolated from various areas of the adult brain and spinal cord.

### Foetal stem cells

A relatively new stem cell type belongs to the group of foetal stem cells (FSC) [23] which can be derived either from foetus or from extra embryonic structures of foetal origin. Foetal stem cells do not form teratomas. Various subtypes of foetal stem cells have been described based on the tissues from which they are derived (i.e. amniotic fluid, umbilical cord, Wharton's jelly, amniotic membrane and placenta). The relatively easy accessibility and high proliferation rate makes foetal stem cells ideal sources for regenerative medicine. Considering their features foetal stem cells can be considered a developmental and operational intermediate between ESCs and SSCs [24-27].

### Mesenchymal stem cells

The first clinical trials with adult stem/progenitor cells to repair non-haematopoietic tissues were carried out with MSCs [28]. The initial clinical trials with MSCs were in osteogenesis imperfecta patients [29] and in patients suffering mucopolysaccharidoses [30]. Other indications for which clinical trials using SSC have been initiated are suppression of GVHD severe autoimmune diseases, repair of skeletal tissue, amyotrophic lateral sclerosis, chronic spinal cord injury, non-healing chronic wounds, vascular disease, coronary artery disease and myocardial infarction. Currently, the largest number of clinical trials is in patients with heart disease with MSC [28].

Most clinical trials studying stem cell therapy have used MSC which were often derived from bone marrow [31,32]. This large interest in MSC applicability for clinical approaches relies on the ease of their isolation from several human tissues, such as bone marrow, adipose tissue, placenta, and amniotic fluid, on their extensive capacity for in vitro expansion (as many as 50 population doublings in about 10 weeks) and on their multipotential differentiation capacity (osteoblasts, chondrocytes and adipocytes) [32-34].

## Risk factors

Risks associated with stem cell therapy depend on many risk factors. A risk is defined as a combination of the probability of occurrence of harm and the severity of that harm [35,36]. A risk factor or hazard is defined as a potential source of harm [35,37]. Examples of risk factors are the type of stem cells used, their procurement and culturing history, the level of manipulation and site of injection. Because of the variety of risk factors, the risks associated with different stem cell based medicinal products may differ widely as well. For an adequate benefit/risk assessment of a stem cell based medicinal product, all important identified risks (i.e. risks or adverse events identified in clinical experience) as well as potential/theoretical risks (e.g. non-clinical safety concerns that have not been observed in clinical experience) [38] should be thoroughly evaluated. Such an evaluation at the start and during the development of a stem cell based therapy may help to determine the extent and focus of the product development and safety evaluation plans. Here we discuss several risks associated with stem cell based medicinal products, and the risk factors contributing to these risks.

Different categories of risk factors can be distinguished (Table 2). Firstly, risk factors associated with the intrinsic cellular properties of a particular cell type or class of stem cells (Table 1); secondly extrinsic risk factors introduced by procurement, handling, culturing, or storage of the cells; and finally the risk factors associated with the clinical characteristics (e.g. surgical procedures, immunosuppression, site and mode of administration, or co-morbidities) will be discussed. It is important to realize that multiple risk factors from these different categories can contribute to the risk to the patient. In principle, knowledge on potential risks and risk factors obtained with other/existing stem cell based medicinal products may contribute to the risk evaluation of new stem cell based therapies.

**Table 2:** Overview of risk factors and risks associated with stem cell-based therapy

	Risk factors or hazards	Identified risks
Intrinsic factors	- Origin of cells (e.g. autologous vs. allogenic, diseased vs. healthy donor/tissue)	- Rejection of cells
Cell characteristics	- Differentiation status	- Disease susceptibility
	- Tumourigenic potential	- Unwanted biological effect (e.g. in vivo differentiation in unwanted cell type)
	- Proliferation capacity	- Toxicity
	- Life span	- neoplasm formation (benign or malignant)
	- Long term viability	
	- Excretion patterns (e.g. growth factors, cytokines, chemokines)	

Extrinsic factors Manufacturing and handling	- Lack of donor history	- Disease transmission
	- Starting and raw materials	- Reactivation of latent viruses
	- Plasma derived materials	- Cell line contamination (e.g. with unwanted cells, growth media components, chemicals)
	- Contamination by adventitious agents (viral/bacterial/mycoplasma/fungi, prions, parasites)	- Mix-up of autologous patient material
	- Cell handling procedures (e.g. procurement)	- neoplasm formation (benign or malignant)
	- Culture duration	
	- Tumourigenic potential (e.g. culture induced transformation, incomplete removal of undifferentiated cells)	
	- Non cellular components	
	- Pooling of allogenic cell populations	
	- Conservation (e.g. cryopreservatives)	
	- Storage conditions (e.g. failure of traceability, human material labelling)	
	- Transport conditions	

Clinical characteristics	- Therapeutic use (i.e. homologous or non-homologous)	- Undesired immune response (e.g. GVHD)
	- Indication	- Unintended physiological and anatomical consequences (e.g. arrhythmia)
	- Administration route	- Engraftment at unwanted location
	- Initiation of immune responses	- Toxicity
	- Use of immune supressives	- Lack of efficacy
	- Exposure duration	- neoplasm formation (benign or malignant)
	- Underlying disease	
	- Irreversibility of the treatment	

The potential risk of tumour formation will be discussed first. As multiple factors may contribute to tumour formation the risk of tumour formation will be discussed along the lines of these individual factors that are discussed in separate paragraphs. Second are the risks associated with immune responses, particularly for allogeneic stem cell transplantation. Third, is the risk of human pathogen transmission and adventitious agents. Finally, there may be potential other risk factors with yet unknown risks to patients.

### Tumour formation

Stem cell features resemble some of the features of cancer cells, such as long life span, relative apoptosis resistance and ability to replicate for extended periods of time [39,40]. Therefore stem cells may be considered potential candidates for malignant transformation. In addition, similar growth regulators and control mechanisms are involved in both cancer and stem cell maintenance [39]. This is probably why tumour formation is often seen as a key obstacle to the safe use of stem-cell based medicinal products.

The potency of the stem cells (pluri- or multipotent) is an essential factor contributing to the risk of tumour formation (Table 1). However the tumourigenic potential of stem cell based medicinal products also depends on other intrinsic and extrinsic risk factors (Table 2), such as the site of administration (i.e. the local environment of the stem cell in the recipient) and the need for *in vitro *culturing. The manipulation of the cells may also contribute to the tumourigenic potential.

Recently a 13 year-old male ataxia telangiectasia patient was diagnosed with a donor derived multifocal brain tumour 4 years after receiving neural stem cell transplantation. The biopsied tumour was diagnosed as a glioneuronal neoplasm. Analysis showed that the tumour was of non-host origin suggesting it was derived from the transplanted neural stem cells. Microsatellite and HLA analysis demonstrated that the tumour was derived from at least two donors [41]. The neural stem cells used were derived from periventricular tissue from fetuses aborted at week 8-12. The cell population was used after 3-4 passages with the total length of culturing within 12-16 days. 50-100 × 10^6 ^cells, obtained from 1-2 fetuses were given in each treatment in 2-3 cc, either by direct injection into the cerebellar white matter by open neurosurgical procedure or by injection into the patient's CSF by lumbar puncture. Although only karyotypically normal fetuses were used for isolation and preparation of fetal neural stem cells details of the cells after culture are lacking. This anecdotal case report illustrates that the risk of tumour formation of stem cell is not theoretical and should be carefully considered.

#### Cellular characteristics and multi/pluripotency

Risk evaluation regarding the use of pluripotent stem cells (ESC or iPSC) should by definition include the possible occurrence of teratomas (one of the hallmarks of pluripotency). In animal models, not only benign teratomas but also malignant teratocarcinomas have been observed following administration of human ESCs or mouse iPSCs [9,42]. *In vitro *differentiation of ESC/iPSC into specific cell types is preferred as this will reduce the potency of the cells and may thus reduce the risk of tumour formation. However it should be noted that *in vitro *culture should also be considered a tumourigenic risk factor (see discussion below)

Autologous SSC may play a role in the aetiology of cancer where these cells may become tumourigenic [43]. According to this cancer stem cell theory, only small fraction of cells within a tumour, the so-called cancer stem cells, are capable of independent growth, and fulfil the criteria described for (cancer) stem cells [39] (e.g. colony growth in soft agar *in vitro *or in spleen *in vivo *[44,45]. These cancer stem cells have metastatic potential, form tumours in secondary hosts and are believed to be responsible for continuous renewal of cells within the tumour mass. However, despite the similarities between somatic stem cells and cancer stem cells (self renewal, asymmetric division and relative slow proliferation) a direct link between somatic stem cells and cancer stem cells remains to be shown.

Multipotent, unaltered (non-cultured or differentiated) SSC cells have been used extensively in the clinic for decades. HSC are widely used for reconstitution of immune function [20]. Also bone marrow derived mesenchymal stem/stromal cells (MSC) have been used as supportive treatment in HSC transplantation. The clinical experience with these therapies indicates that the i.v. administration of SSC did not reveal major health concerns, and is generally not accompanied by tumour formation. However, limitations of the safety database (i.e. number of patients treated) and lack of long-term follow-up required to study potentially rare adverse events should be taken into account when evaluating the tumourigenic potential of SSC. Autologous bone marrow derived stem cells have been identified as the cell of origin of *Helicobacter*-induced gastric cancer in a mouse model [39,46]. Also osteogenic sarcoma has been reported to originate from a mesenchymal stem cell [47]. In addition, donor-derived cells have been shown to give rise to post-transplant Kaposi sarcoma [48], skin carcinoma [49] and oral squamous cell carcinoma [50]. Notably the incidence of solid tumours is significantly increased in patients that have received a bone marrow transplantation [51], and also recipients of solid organ transplants appear to have a higher incidence of secondary malignancy [39]. Supporting evidence is still lacking if the tumour is caused by the (co-) administered stem cells or by other aspects of the treatment (e.g. immune suppression, radiation or chemotherapy). Therefore also for SSC tumourigenicity may still be a concern, especially when these cells are used for other purposes than haematopoietic reconstitution.

#### Site of administration

The local environment in which the stem cell resides may influence its tumourigenic potential. Removal of the cells from the context of a developing embryo and enforcing *in vitro *culture has been proposed as the cause for the increased tumourigenic potential of ESC when compared to the originator cells (the inner mass of early blastocysts)[43]. The site of human ESC administration in SCID mice is an important factor determining the rate of teratoma formation [52]. In mouse it has been shown that tumourigenicity of (mouse) ESC depends on the host/species to which the cells are administrated. When transplanted into a homologous species mESC caused highly malignant teratocarcinomas at the site of administration, while xenotransplantation in rats resulted in migration and differentiation of the mESC [53]. Similar observations have been reported for human ESC by Shih et al. [42]. More aggressive tumour growth was seen when hESC were injected in human foetal tissue engrafted in SCID mice, while differentiated teratomas were formed when these cells were injected directly in SCID mouse tissue.

#### *In vitro *stem cell culture

For ESC and iPSC in vitro differentiation is a requirement for clinical application as these cells are inherently tumourigenic when they are in their pluripotent state. Also for SSC *ex vivo/in vitro *proliferation and/or differentiation of stem cells prior to administration to a patient may be desirable in certain circumstances.

*In vitro *expansion and culture of stem cells can change the characteristics of the stem cell due to intracellular and extracellular influences. Every cell division has a small chance of introducing deleterious mutations and mechanisms to correct these alterations may not function as adequate (e.g. cell cycle arrest, DNA repair), or at all (e.g. immune recognition) occur during *in vitro *culture. Cell culture induced copy number changes and loss of heterozygosity have been reported for hESC lines [54]. In principle, such changes may cause transformation of a cell into a tumourigenic phenotype and may contribute to increased tumour formation. The clinical relevance (with regard to tumourigenic potential) of these alterations (e.g. chromosomal aberrations) still remains a matter of debate [40]. Some reports indicated that the tumourigenicity of stem cells has been predicted to increase proportionally with the length of in vitro culturing [43]. In vitro ESC lines have been reported to show a certain degree of deregulation of the so-called imprinted genes, also after differentiation [20].

Spontaneous malignant transformation of mouse MSC following long term *in vitro *culture has been described [55-57]. Also spontaneous transformation of mice neural precursor/stem cells has been reported [58]. These transformed cells were detected already after ~10 passages of cell culture, and produced tumours *in vivo *upon administration into rodent brains.

Transformation of human MSC has also been investigated. No supporting evidence for transformation of human MSC has been found independently by several authors, even after extensive genetic characterisation [59-61]. Some publications have reported spontaneous transformation of human MSC [62,63]. However, several of these authors have reported that the occurrence of transformed cells in their human MSC culture was due to cross contamination of the original cell culture with tumour cells [64-66]. There is therefore still controversy whether, similar to mouse, also human MSC can transform into a malignant cell type after in vitro culture. Chromosomal alterations have been observed in MSC cultures [64,67] including in clinical grade cultures [61,67]. These karyotipic alterations often seem to concern aneuploidy [64], of chromosome 5 in particular and to a lesser extent of chromosome 8 and 20. It was suggested that the occurrence of aneuploidy could be donor dependent [61]. Interestingly, the abnormal karyotype did not always persist upon prolonged culturing [68]. Due to the delay in karyotype analysis, in a few cases MSC with karyotypic alterations have been injected into human recipients and no tumour formation has been observed (up to 2 years follow up) [61]. Nevertheless, very limited patient numbers could explain limited number of observations.

It may therefore be concluded that although chromosomal aberrations have been observed after in vitro culture of MSC, spontaneous in vitro malignant transformation is still a matter of debate. At the moment, human MSC appear to be less prone to malignant transformation during in vitro culture when compared to murine MSC [61,69], but further studies are urgently needed.

#### Genetic modification

Some stem cells (e.g. iPSC) may require extensive manufacturing steps, including genetic modification/reprogramming prior to their clinical application. It is important to consider the different methods available to generate iPSCs as depending on the used methodology specific risk factors can be relevant.

Retroviruses and lentiviruses have been used to generate mouse or human iPSCs. These viruses were genetically altered to encode the genes that are required for transformation into an iPSC. Applying this genetic reprogramming, the used viruses can integrate into the cell genome. Consequently the cells may contain multiple viral integration sites in their genomes. The use of retroviruses and lentiviruses raises safety issues similar to those that have been observed in the gene therapy of patients with X-linked severe combined immunodeficiency for which the occurrence of cancer has been reported due to integration of therapeutic vectors activating oncogenes [70,71]. It should be noted that in iPSC generation this risk factor may be controlled as viral integration sites can be determined in iPSC clones, which enables exclusion of clones that show unwanted (i.e. potentially hazardous) integration. A second risk factor involved with the use of retroviruses and lentiviruses is transgene reactivation. The reactivation of one of the reprogramming factors, *c-Myc*, may result in tumour formation which has been observed in approximately 50% of chimeric mice generated from iPSCs [9]. It has been demonstrated that using a Cre-mediated strategy iPSCs have been generated by genomic integration of the reprogramming factors which were removed from the genome by excision or transposase activity. Consequently the negative effect related to the integration of the reprogramming factors is prevented [72-74].

Viral integration and the use of oncogenes is not the only risk factor that may lead to tumour formation following the generation of iPSCs. iPSC induction is also associated with profound and progressive changes in the epigenetic state of the chromatin [75]. Epigenetic changes have been suggested to change the tumourigenic potential of cells, e.g. by changing in the expression of oncogenes or tumour suppressor genes. However there is currently not enough data for evaluating the possible contribution of epigenetic changes to the risk of tumour formation. Also reactivation of other (host) reprogramming factors may cause tumour formation. Furthermore it has been suggested that sustained expression of the reprogramming transgenes might suppress differentiation of iPSCs which may result in an increased tendency to teratoma formation when these cells are transplanted into patients [76].

Two other strategies are developed to generate iPSCs with a reduced risk of tumour formation while viral integration is prevented. Firstly, induction of iPSCs has also been achieved without viral integration using adenoviral vectors [77] or plasmids [78] that encode the required reprogramming factors. Secondly chemicals and small molecules have been used successfully to generate iPSCs. These methods are based on the endogenous activation of reprogramming factors as was reported for the reactivation of the Oct3/4 gene [79,80]. However, it should be noted that even in the absence of transgene integration, small plasmid fragments may integrate or chemically induced mutations could occur. Depending on the integration site or mutation characteristics other negative effects may be observed [76].

Taken together, the knowledge on iPSC is expanding rapidly and the methods to generate them may have decrease the risks associated with their generation (e.g. associated with use of retroviruses), yet there is still very limited data on the tumourigenicity related risks of iPSC.

#### Bystander tumour formation

In addition to be tumour forming cells themselves, stem cells might affect the growth/proliferation of existing tumour cells [28]. This has been studied for MSC only. *In vitro *and *in vivo *studies have reported inhibition, enhancement and no effect of administration of MSC on tumour growth [81-85]. Most likely the observed effect depends on the nature of the cancer cells, the characteristics of the used MSC, on the integrity of the immune system and on the timing and site of injection. Two possible mechanisms have been postulated for the stimulation of tumour growth [82], MSC may provide supportive stroma creating a permissive environment for tumour growth or MSC may reduce immune rejection (see section on immune modulation below) of the tumour cells thus allowing continued tumour growth. No mechanism for the sometimes observed decreased tumour growth has been postulated. Since all these studies have been performed *in vitro *or in animal models the relevance of these observations for the clinical use in humans is unknown. Notably, an opposite effect on tumour growth, between *in vitro *and *in vivo *situation, has also been reported [81] and has complicated the assessment of the potential effect of MSC on tumour growth. Thus, potential risk of stimulation of growth of a previously undetected tumour by MSC must be considered when administering these cells to a patient; however the likelihood of this risk is difficult to assess.

Options to mitigate the risk on tumourigenicity include the induction of differentiation, possibly accompanied by cell sorting to minimise the number of pluri/multipotent stem cells in the cell preparation [86], or to separate tumourigenic stem cells from non-tumourigenic stem cells (by e.g. cell sorting on specific 'tumourigenic' surface antigens). It should be noted that, in practice, finding truly specific antigens for selection the desired cell population may be challenging. Another approach would be to selectively kill unwanted/stem cells by e.g. the introduction of a suicide gene, the generation of killer antibodies specific for stem cell surface antigens, or chemotherapeutic treatment (hESC and iPSC are fast growing cells).

### Immune responses

Administration of stem cells may affect the host immune system. The administered cells may directly induce an immune response [86] or may have a modulating effect on the immune system.

Both ESC-derived cells [87-89] and especially MSCs [81,90,91] have been reported to be immune-privileged and have a low immunogenic potential. Consequently allogenic administration may require reduced or even no immune suppression. However, upon differentiation these cells may become more immunogenic due to e.g. upregulation of a normal set of MHC molecules. Especially in case of cells that are not intended to be used for the same essential function or functions in the recipient as in the donor (non-homologous use) or when administered at non-physiological sites, immunogenicity of the cells may alter and thus remains unpredictable.

Immune recognition of the administered cells is particularly important when the cells are non-autologous. Evidently, careful HLA-matching of donor and recipient may diminish the risk on Graft-versus-Host disease (GVHD), but is often not readily achievable.

Graft rejection may lead to loss-of-function of the administered cells and consequently compromise therapeutic activity. The use of immune suppressants may limit this risk, but may elicit drug related adverse reactions. Other strategies to prevent immune rejection of the transplanted cells have been proposed and could include banking ESC, iPSC or even SSC cells with defined major histocompatibility complex backgrounds or genetically manipulating the stem cells to reduce or actively combat immune rejection [3,92].

The immune modulatory effect of both ESC and MSC has been described in multiple reports, mostly describing *in vitro *experiments. MSC have been described to suppress T cell proliferation, inhibit differentiation of monocyte and cord blood CD34^+ ^cells into immature myeloid DC, affect DC function (skewing mature DC towards immature state [90], inhibit TNF production, increase IL-10 production), and inhibit proliferation and cytotoxicity of resting NK cells and their cytokine production [65]. A direct effect of MSC on B cells is still matter of debate (conflicting results), however most studies indicate that MSC can inhibit B cell proliferation and/or differentiation *in vitro *[65]. Recently, in vitro studies have demonstrated that both human and mouse ESC extracts retain the immune modulatory properties of ESCs and ESC derived factors can inhibit human mDC maturation and function [89].

*In vivo *control or limitation of GVHD by MSC has been reported both in humans [93] and animal models [81,83]. In a small clinical study, MSC cotransplantation with HSC of HLA-identical siblings the observed decreased frequency in GVHD (acute and chronic) was accompanied by an increased frequency of relapse of the treated of haematological malignancy [83].

An immune suppressive effect of MSC has also been observed in an animal model of rheumatoid arthritis [90]. In addition, MSCs have been shown to suppress lymphocyte proliferation to allogenic or xenogenic antigens [81,82,84] leading to acceptation of allo/xenotransplants in animal models [90]. In clinical studies MSC have been used to facilitate the engraftment of HSC and decrease GVHD [81].

Taken together the *in vitro *and some *in vivo *data suggest that MSC can interact with cells of both the innate and adaptive immune system and can modulate their effector functions leading to potent immunosuppressive and anti-inflammatory effects. The secretion of various soluble factors by MSC [84] may enhance this effect. It has been described that MSC express Toll like receptors (TLRs) that after interaction induce proliferation, migration and differentiation of the MSC and the secretion of cytokines [81]. MSC may thus exert protective effects resulting in e.g. effective stimulation or regeneration of cells *in situ *or in a local immunosuppressive microenvironment. Knowledge regarding mechanisms by which MSC or ESC-derived cells exert their immune suppressive effect is still increasing [81]. Nevertheless, the extrapolation from animal or *in vitro *studies to human is relatively unpredictable and both beneficial and adverse effects should be considered.

### Adventitious agents

Manufacturing of cell based medicinal products inevitably does not include terminal sterilization, purification, viral removal and inactivation. Therefore, viral and microbial safety is a pivotal risk factor associated with the use of non-autologous and/or cultured cells, including stem cells. These risk factors are not unique to stem cells and apply to all cell based medicinal products. Donor history is of particular importance for stem cell lines which were initially intended for research purposes, rather than to be used in clinical application. The risk of donor-to-recipient transmission of bacterial, viral, fungal or prion pathogens may lead to life-threatening and even fatal reactions. Disease transmission has been reported after allograft transplantation [94,95]. Only limited information is available on disease transmission via adult somatic stem cells other than those routinely used HCS. It has been shown that MSC are susceptible to both CMV and HSV-1 infection *in vitro*. However, using sensitive PCR techniques no CMV DNA could be detected in *ex vivo *expanded MSC derived from healthy CMV positive individuals [96]. No information on the susceptibility for adventitious agents of pluripotent stem cells has been reported in the scientific literature.

Although progress has been made in tissue culturing techniques, both serum and feeder layers are occasionally still needed for the *in vitro *isolation and propagation of (pluripotent and somatic) stem cells [91], The use of animal products in tissue culture (e.g. foetal bovine serum (FBS), or non-human feeder cells) also may introduce a risk of transmission of disease (*e.g*. prion) as well as activation of host immune system by biomolecules [97] (e.g. non-human sialic acid) [69]. Expansion of stem cells in medium supplemented with FBS has a potential risk of transmitting viral and prion diseases and causing immunological rejection. Autologous or donor-derived plasma may be a safer substitute for FBS and may still allow proper cell proliferation and differentiation. In fact, changing FBS to human platelet lysate has been described to result in accelerated/enhanced proliferation, without genetic abnormalities [69]. However, the use of autologous patient serum may be less favourable because serum derived from aged individuals has been reported to interfere with MSC proliferation and differentiation capacity [69]. When possible, cell feeder free isolation and culturing or the use of a membrane between feeder cell and stem cell culture will enhance the viral safety of the stem cell based medicinal product.

Most of the ESC lines used today have been generated for basic research, with the application in humans not yet in mind. These cell lines have not been isolated under FBS- and feeder cell free conditions. Now clinical application for some of these ESC lines may be dawning, and potential contaminations with adventitious agents becomes a safety issue that should be thoroughly addressed. However, because each individual ESC line can be considered as unique, 'simple' regeneration of an ESC line under safer culturing conditions is not always readily achieved.

Testing for adventitious agents will increase the safety of stem cell based medicinal products. This may be feasible for products where the number of cells is not limited, for example for ESC or iPSC cell lines with indefinite self-renewal capacity. However for individually prepared cell batches or SSC preparations there may not be sufficient material to both test for the presence of adventitious agents and to treat the patient(s).

Another aspect of viral safety is the patient's vulnerability to the contraction or reactivation of (latent) viruses due to immune suppression necessary for some types of stem cell therapy. In the case of allogenic stem cell therapy the use of immune suppressive agents may be required leading to a (severe) compromised host immune system. In HSC transplantation, allogeneic stem cell transplantation is often complicated by reactivation of herpes viruses indicating that viral activation is not only a theoretical risk.

### Other risk factors

There are several other risk factors which need to be considered before the clinical application of (stem) cells. For most of these factors only limited scientific evidence is available.

#### Biodistribution/Ectopic grafting

An important risk factor is the (bio)distribution of the administered stem cells. MSC are known to home to specific tissues e.g. the bone marrow, muscle, or spleen, particularly when the tissues are damaged or under pathological conditions such as ischemia or cancer [32,81,82,84,85]. The mechanism underlying the migration of MSC remains to be clarified. Data suggests that both chemokines and their receptors and adhesion molecules are involved. However, it has been reported that when used to treat myocardial infarction (MI) only a few cells homed to the site of injury following intravenous administration, and engraftment rate appears to be extremely low even when injected at site of injury (intramyocardial or intracoronary injection) [17,98]. It is unclear where the non-engrafted (stem) cells go to, and also the risks associated with distribution to undesired tissues are unknown. One possibility is the engraftment of the stem cells at these distant or non-target sites. As noted earlier the local environment in which the stem cell resides in the recipient may influence the biological properties of the stem cells, however only little is known whether these effects are potentially harmful or not. Given the limited data, the risk of such ectopic engraftment and its effects remains unpredictable and should be taken into account.

#### Mode and site of administration

Another risk factor associated with the use of stem cells may be the potential high number of cells needed for the beneficial effect. It is generally unknown how many cells are needed, however, given the (very) low rate of retention and possible low cell survival, large number of cells may be required for obtaining maximal clinical benefit. Injection of concentrated cells into tissue may have unwanted effects. Cells may form aggregates, particularly if sheared by passage through small needles [28]. These aggregates could cause pulmonary emboli or infarctions after infusion. Injection in the portal vein may partially circumvent this problem; however this requires specialised (surgical) procedures which may introduce other risks. Serious adverse events due to procedural complications in combination with underlying disease conditions (e.g. veno-oclusive disease) have been reported during clinical experience with HSC transplantation [99,100].

Similarly, application of the cells at specific locations (e.g. site of injury) may be desirable, e.g. intracardial, at site of spinal cord injury or brain lesion, but also for this specific procedures and/or surgery may be required with associated risks.

#### Unwanted (de)differentiation

As mentioned before, it is unlikely that undifferentiated iPSC or ESC will be used in the clinic, and that in vitro differentiation into a desired phenotype will be necessary prior to administration. However, it is unknown if dedifferentiation of stem cells can occur *in vitro *or *in vivo*. Dedifferentiation of somatic cells or redifferentiation into another cell type has been described [20], whether this has adverse clinical consequences remains unclear. In addition, for MSC differentiation into unwanted mesenchymal cell types such as osteocytes and adipocytes has been described [101]. Encapsulated structures containing calcification and/or ossifications in the heart have been seen in animals treated with BM-derived MSC for (induced) myocardial infarction [101]. It can be concluded that unwanted differentiation is therefore not only a theoretical risk; however the factors contributing to this risk are unknown. Differentiation or culturing of stem cells could not only induce malignant transformation but in theory may also induce cellular alterations such as altered excretion patterns or cell surface molecules which may influence the *in vivo *attributes of the administered cells. This may have unexpected adverse or toxic consequences.

#### Non-homologous use

Although the use of MSC and HSC have a excellent track record in some routine clinical applications (bone marrow transplantation or reconstituting of immuno-depleted patients) many of the potential risks discussed above (e.g. ectopic grafting, unpredictable immune consequences, (de)differentiation) can also be relevant to these type of cells in case they are not intended to be used for the same essential function or functions in the recipient as in the donor (so-called non-homologous use). The potential unpredictable adverse effects clearly need further evaluation.

#### Purity and identity

Another critical issue to address is the need for obtaining a pure population of the desired stem cells. Contamination with other types of cells could cause undesirable effects [102], or in case of ESC derived cells undifferentiated ESC could be a potential source for tumour formation. In addition, several publications reporting on MSC to undergo spontaneous transformation events have recently been retracted since the reported observations could not be reproduced [64,65,103]. It was confirmed that the initial observations were based on cross contaminated HT1080 human fibrosarcoma cells. Obviously such errors should be preventable by Good Manufacturing or Good Laboratory practices but these examples illustrate that even relatively simple risks should be considered.

#### (Lack of) functional characteristics

There may also be risks associated with specific stem cell therapies. An example is the use of stem cell therapy in the treatment of myocardial infarction (MI). One of the main safety concerns is the occurrence of arrhythmias [98,104]. These were seen in some, but not all trials using stem cell-based therapy in treatment of heart failure or myocardial infarction [98]. The used cell type and route of administration may influence the risk on arrhythmias [98]. These arrhythmias may be caused by poor cell-cell coupling, incomplete differentiation (seen *in vitro *with MSC), an unexcitable state of the MSC, or a heterogeneous distribution of action potential [98,104]. Principally, cell therapy in the heart can be predicted to have a multitude of electrical effects some potential destabilizing and others clearly beneficial.

#### Donor and recipient clinical characteristics

Evidently, if allogenic stem cells are used there is a risk of stem cell-tissue rejection which may be (partially) overcome by donor-patient matching, by immunological sequestration or by the use of immune suppressants, which all have their own drawbacks.

Numerous other factors can be identified which may or may not contribute to a risk associated with the clinical application of a stem cell based medicinal product. These may be specific intrinsic characteristics of the stem cell based medicinal product or more extrinsic risk factors related to e.g. the manufacturing or type of application of the product. For example, when used in an autologous setting, the underlying disease, or medication may have an impact on the number and functionality of the stem cells [34,105], which can induce unwanted side effect of stem cell therapy. Another example may be the (unknown or unidentified) secretion of trophic factors and/or a variety of growth factors by the stem cells [32].

## Conclusion

Initial clinical experience with somatic stem cell therapy may appear promising. However, many questions regarding the potential risks have not yet been answered. The amount of data and the knowledge of risks associated with the use of stem cell therapy are expanding. However, due to the large variation amongst the studies (e.g. study protocol, patient population, heterogeneity of the administered cell population, timing/location of injection) it is difficult to extrapolate results from one study to another, and also from one stem cell based medicinal product to another. Currently, the most extensive clinical experience has been obtained with haematopoietic stem cells and mesenchymal stem/stromal cells. The clinical experience with endothelial progenitor cells is also growing.

In most cases, irrespective of the treated condition or mode of administration, MSC therapy appears relatively safe [31,33,98,106]. However given the limited time of follow up, the low number of patients, the variation in cell preparations and characterisation and mode of delivery, further studies on the safety of MSC are still needed, especially on long term effects such as tumourigenicity. Autologous stem cell transplantation is perceived as non-harmful; however this only applies for non-substantially manipulated stem cells. The risks associated with autologous stem cells that are substantially manipulated (e.g. by tissue culture or genetic modification) or cells that are not intended to be used for the same essential function or functions in the recipient as in the donor do need further evaluation.

In contrast to SSC, there is currently no clinical experience with pluripotent stem cells. This is in particular due to the assumption that the application of these cells is associated with a higher risk in particular related to tumourigenicity. Recent developments indicate that clinical experience with embryonic stem cells become available in the near future. At this moment we are aware of 3 clinical trials using human ESC-derived cells that have been approved by the FDA. The first approved trial is using oligodendrocyte progenitor cells aimed at the treatment of spinal cord injury. This trial has been temporarily put on hold before the first patient was included due to non-clinical findings of microscopic cysts in the regenerating injury site [107]. However the hold has been lifted and recruitment is currently ongoing. The two other trials have just been cleared by the FDA, and aim to treat two eye diseases (Stargardt's macular dystrophy and dry age-related macular degeneration) with ESC-derived cells http://www.advancedcell.com.

As discussed earlier, the perceived risk on tumour formation is higher for iPSC than for ESC. Clinical application of iPSC is still relatively far away as the technique to generate these cells is still quite new and the methods to generate these cells more safely are rapidly developing. For iPSC, even the non-clinical information on the tumour formation in a context relevant to regenerative medicine (focal injection or iv administration) is still very limited (mouse iPSC) or essentially lacking (human iPSC) apart from their teratoma inducing capabilities [43].

Overall stem cell therapy may represent great hope for multiple diseases and degenerative conditions, but a thorough evaluation of the risk factors and potential risk of a stem cell based medicinal product must be a prerequisite step before wider clinical application and/or registration can be accepted. For each stem cell based medicinal product the potential risks to the patient needs to be adequately evaluated and should take into account not only the specific intrinsic characteristics of a specific stem cell but also the safety data already obtained with similar type of products. In addition extrinsic risk factors like manufacturing, handling, storage- and clinical or treatment related risk factors can contribute to the overall risk to the patient. During the risk evaluation, knowledge of the safety of (similar) stem cell based medicinal products may be of great value. Documented/identified risks, and known risk factors as well as potential/theoretical risks should be considered in the risk evaluation. Table 2 presents a (non-exhaustive) overview of risk factors and risks. It should be clear that while tumour formation is an important risk associated with stem cell therapy, other risks (e.g. adverse immune modulation) as well as strategies to minimize the risks should be should be carefully evaluated [35,38,108].

Furthermore, for the successful development of a stem cell based medicinal product more information on the biological mechanism of stem cell therapy is needed as well as sufficient characterisation of the cells and reproducible production of stem cell batches [109]. The current knowledge on the mechanism of action of stem cell therapy is still limited and the cellular requirements necessary for a successful product are largely unknown [34,109]. Other issues such as choice of stem cells to be used, the need/possibility for concurrent tissue regeneration in case of irreversible tissue loss, the differentiation degree and specific identity of the transplanted cells, and the long-term survival of engrafted cells in the absence of a normal supportive tissue environment should be considered as well.

## List of abbreviations

BM: bone marrow; DC: dendritic cells; EPS: endothelial progenitor cells; ESC: embryonic stem cells; FBS: foetal bovine serum; GVHD: graft versus host disease; HLA: human leukocyte antigen; HSC: Haematopoietic stem cells; iPSC: induced pluripotent stem cells; LVEF: left ventricular ejection fraction; MHC: major histocompatibility complex; MI: myocardial infarction; MSC: mesenchymal stem/stromal cells; SSC: somatic stem cells; TLR: toll like receptor.

## Competing interests

All authors declare no competing interest. The views expressed in this article are the personal views of the author(s) and may not be understood or quoted as being made on behalf of or reflecting the position of the Netherlands Medicines Evaluation Board.

## Authors' contributions

All authors contributed to the writing and discussion of the manuscript.

The views expressed in this article are the personal views of the authors.
